# Exploring the Association of VEGF-A and ANGPTL2 with the Prognosis of Non-proliferative and Proliferative Diabetic Retinopathy

**DOI:** 10.7759/cureus.68273

**Published:** 2024-08-31

**Authors:** T Shakthiya, Leena Chand, Radha Annamalai, Arul Senghor K A

**Affiliations:** 1 Biochemistry, Sri Ramachandra Institute of Higher Education and Research, Chennai, IND; 2 Ophthalmology, Sri Ramachandra Institute of Higher Education and Research, Chennai, IND; 3 Biochemistry, SRM Medical College Hospital and Research Centre, Chennai, IND

**Keywords:** angiopoietin-like protein-2, enzyme-linked immunosorbent assay (elisa), diabetic retinopathy, fasting blood glucose, vascular endothelial growth factor

## Abstract

Introduction

Diabetic retinopathy (DR) is a microvascular ailment that can arise from the long-term effects of diabetes mellitus. It can potentially cause retinal damage that could endanger vision and cause blindness. The worsening of DR is mainly linked to poor glycemic control, uncontrolled hypertension, and dyslipidemia. There is a need for alternative and clinically significant novel molecules involved in the pathogenesis of DR because the diagnostic and prognostic markers have reached a limit.

Materials and method

This study included sex and age-matched diabetic patients with proliferative stage (N = 70), non-proliferative stage (N = 80), and control (N = 80, without the sign of DR). These patients were recruited from outpatients in the Department of Ophthalmology, Sri Ramachandra Institute of Higher Education and Research, Chennai, India. A random blood sample was collected from each study participant, and the serum was separated after centrifugation and stored at -80 °C for batch analysis. The biomarkers vascular endothelial growth factor (VEGF-A) and angiopoietin-like protein-2 (ANGPTL2) were measured using a sandwich enzyme-linked immunosorbent assay (ELISA) technique, and the laboratory parameters such as fasting blood sugar (FBS), lipid profile, blood urea nitrogen (BUN), creatine, and glycated hemoglobin (HbA1C) were also assessed.

Results

We observed statistically significant differences in the duration of diabetes, FBS, total cholesterol (TC), triglyceride level (TGL), BUN, and creatine (p<0.05), and the mean age of study participants was 52.95±8.20 years in the control group, 53.85±10.20 years in the proliferative diabetic retinopathy (PDR) group, and 55.02±7.65 in the non-proliferative diabetic retinopathy (NPDR) group. Furthermore, ANGPTL2 levels were statistically significant according to the severity of the disease (p<0.001*), and they were also linked (p<0.05) with established markers such as VEGF-A.

Conclusion

Thus, our research implies that the up-regulated markers might be linked to the disease's advancement and could serve as a prognostic indicator or therapeutic target for DR.

## Introduction

Hyperglycemia-induced retinal vascular disease, known as diabetic retinopathy (DR), is the most prevalent microvascular complication of diabetes and a major contributor to avoidable blindness and visual impairment [1]. The number of people suffering from DR is increasing gradually and is expected to reach 191 million by 2030 [2]. Due to the rising global and national incidence of this illness, a cost-effective and affordable diagnostic test is becoming increasingly important to prevent or delay the disease’s progression into the advanced proliferative stage and to prevent blindness.

Vascular endothelial growth factor (VEGF-A) is the main regulator of ocular angiogenesis since it has been linked to the onset of DR and has been effectively treated with anti-VEGF medications in DR patients [3]. The administration of this drug intravitreally has been linked to a decrease in the levels of certain growth factors and pro-inflammatory cytokines produced by mononuclear phagocytes in the intraocular fluid [4] of DR patients. Though using VEGF inhibitors has transformed the management of neovascularization-related retinal illnesses, some do not respond or experience inadequate or suboptimal responses over time [5] and fail to achieve notable visual improvement. This emphasizes how the pathophysiology of proliferative diabetic retinopathy (PDR) and non-proliferative diabetic retinopathy (NPDR) is influenced by the expression of different angiogenic factors in the retinal vasculature, necessitating further investigation. Angiogenesis inhibitors are also effective in treating disorders involving the formation of new blood vessels, such as wet macular degeneration in the eye. 

Angiopoietin-like protein-2 (ANGPTL2) is a secretory glycoprotein produced from adipose tissue that belongs to a member of the angiopoietin-like family. It resembles angiopoietin structurally because it has a coiled-coil domain and a fibrinogen-like domain [6, 7]. It is primarily known for pro-inflammatory activity and is abundantly expressed in the heart, adipose tissue, kidney, lung, and skeletal muscle [8]. It is necessary for the regulation of angiogenesis as well as the metabolism of fat and glucose [9]. It was demonstrated by Horio et al. [10] that endothelial cell-derived ANGPTL2 enhanced vascular inflammation, leading to endothelial dysfunction, atherogenesis, and plaque instability by triggering integrin α5β1/nuclear factor kappa B (NFκB) signaling and augmenting macrophage infiltration. This activation of NF-β signaling worsens endothelial dysfunction and causes retinal neovascularization [9]. 

To improve the risk stratification for vision loss in these patients, analyzing the altered expression of angiogenic factors may be a promising predictive strategy for advanced eye disease. It can also help to identify novel therapeutic targets that define new benchmarks for DR treatment. However, further research is required to fully understand the physiological and pathological functions of these markers in specific retinal diseases.

This study assessed the levels of VEGF-A and ANGPTL2 in the development of DR, and a correlation between markers was observed along with the severity of the disease.

## Materials and methods

Patient recruitment

This descriptive cross-sectional study included 230 participants, with women and men aged between 35 and 80. All participants were recruited from the outpatient Department of Ophthalmology, Sri Ramachandra Institute of Higher Education and Research, Chennai, India, between December 2021 and December 2023. Anthropometric measurements (weight and height) were collected for BMI calculation. The diagnosis of diabetes was based on the criteria of the American Diabetes Association [11]. Clinical information regarding past medical history and diabetes duration was obtained through a standardized inquiry. Hypertension was defined as blood pressure (BP) above 140/90 mmHg or under treatment with antihypertensive drugs. The participants were divided into three groups based on the fundus findings as PDR (n = 70), NPDR (n = 80), and age- and sex-matched type 2 diabetes mellitus (T2DM) with no DR (n = 80) as controls. The exclusion criteria were cataracts at the time of recruitment, cancer, pregnancy, psychological disorders, diabetic nephropathy, hypertensive retinopathy, autoimmune disease, and any other retinal illness like uveitis or glaucoma.

Diagnosis of diabetic retinopathy

To prevent selection bias, a skilled ophthalmologist who was blinded for this study conducted dilated ophthalmic eye examinations using techniques like fundus photography, optical coherence tomography, and slit lamp biomicroscopy. The Early Treatment Diabetic Retinopathy Study (ETDRS) classification was used to evaluate the severity of DR. Individuals with retinal hemorrhages, cotton wool spots, and microaneurysms were classified as NPDR; those with retinal detachment and neovascularization were classified as PDR, and those without retinopathy were taken as the control group [12]. Through a standardized inquiry, clinical data regarding medical history and the duration of diabetes was collected.

Clinical information and laboratory analysis

Information on sex, age, weight, height, duration of diabetes, history of cataracts, comorbidities, and drug history was collected, and informed consent was obtained from the study participants before enrollment. Confidentiality was maintained throughout the study. A random blood sample was collected from each study participant, and the serum was separated after centrifugation. This serum sample was stored at -80℃ for batch analysis. Fasting blood sugar (FBS), lipid profile, blood urea nitrogen (BUN), and creatine were estimated by automated device AU 5800, chemistry (Beckman Coulter, Brea, CA). Glycated hemoglobin (HbA1c) was performed using high-pressure liquid chromatography (HPLC). 

Circulating VEGF-A and ANGPTL2 levels

The levels of VEGF-A were determined using the sandwich enzyme-linked immunosorbent assay (ELISA) kit (elabscience cat no. E-TSEL-H0026) according to the manufacturer's instructions. The ELISA system had a sensitivity of 0.03 ng/mL. Both intra-coefficients of variability (CV) and inter-CV were <10%.

The serum ANGPTL2 was measured using sandwich ELISA (elabscience cat no. E-EL-H6034) following the manufacturer’s instructions. The ELISA system had a sensitivity of 0.10 ng/mL. Both intra-CV and inter-CV are <10%

Sample size

The sample size was determined to detect a medium effect size (d) of 0.5 with an alpha of 0.05 and 80% power. Using OpenEpi software, version 3 (The OpenEpi Project, Atlanta, GA), the estimated total sample size is 160, but it was increased to 230. 

Statistical methods

The data were processed using IBM SPSS software for Windows (version 26, IBM, Chicago, USA). Categorical variables such as gender were expressed as frequency and percentages. Continuous variables such as age, duration of diabetes, and other biochemical assessments were expressed as mean (standard deviation). The normal distribution of the data was checked using the Shapiro-Wilk test. As the data followed a normal distribution, a parametric test was used. For comparisons among multiple groups, a one-way ANOVA followed by Bonferroni post-hoc analysis was conducted. Pearson correlation analysis was done to examine the correlation between serum markers. The stepwise multiple regression model was constructed based on all subjects, considering ANGPTL2 levels as response variables and including high-density lipoprotein cholesterol (HDL-C), total cholesterol (TC), triglyceride level (TGL), and DR stage, which were regarded as explanatory variables for both NPDR and PDR groups. Collinearity was assessed by calculating the variance inflation factor (VIF), and variables with VIF ≥5 were excluded from the models. Binary logistic regression analysis was conducted to detect the independent effect of these markers on the presence of DR, considering DR occurrences as response variables and sex, age, and T2DM duration as covariates. Receiver operator characteristic (ROC) curve analysis was performed to derive cut-off values for the markers in NPDR and PDR.

## Results

Clinical characteristics of the study subjects

There were 230 participants recruited for the study. Of them, 36.9% were female and 63.1% were male. The mean age of study participants was 52.95±8.20 years in the control group, 53.85±10.20 years in the PDR group, and 55.02±7.65 years in the NPDR group. The duration of diabetes among our study participants ranged from one month to 40 years, and 29% of patients had a history of hypertension with NPDR. Patients having cataracts at the time of sample collection were not chosen for the study; however, those who previously had a history of cataracts were included; 5% of the control group, 40% of the NPDR group, and 41.9% of those with PDR had prior cataracts. More patients with PDR received insulin treatment compared with those without. The mean of the fasting glucose, renal profile, and length of diabetes demonstrated statistically significant differences between the three groups (P<0.05). The lipid profiles of TC (p = 0.03) and TGL (p = 0.01*) were statistically significant among these groups. The mean BUN values in control, NPDR, and PDR groups were 11.72±4.26 mmol/L, 13.8±8.79 mmol/L, and 20.91±13.10 mmol/L, and these differences were statistically significant (p-value<0.001). The mean creatine values of control, NPDR, and PDR were 0.89±0.31 սmol/L, 1.15±0.90 սmol/L, and 2.41±0.76 սmol/L, respectively, and this difference was also found to be statistically significant (p-value<0.001). No significant differences were observed in sex, age, BMI, systolic BP, diastolic BP, HDL-C, or LDL-C among the three groups. The study participants’ clinical and demographic information is displayed in Table 1.

**Table 1:** Comparison of general characteristics and biochemical parameters among three study groups Values are expressed as mean± standard deviation or median; *p<0.05: statistically significant DR: diabetic retinopathy; BP: blood pressure; FBS: fasting blood sugar; HbA1C: glycated hemoglobin; TGL: triglycerides; HDL-C: high-density lipoprotein cholesterol; LDL-C: low-density lipoprotein cholesterol; BUN: blood urea nitrogen

Variables	Diabetic without DR	Non-proliferative DR	Proliferative DR	p-value
	Mean ± SD	Mean ± SD	Mean ± SD	
Age (years)	52.95±8.20	55.02±7.65	53.85±10.20	0.91
Sex				
Male	68%	59%	80%	0.89
Female	32%	41%	20%	
Duration of diabetes (years)	6.265±5.01	12.48±6.92	3.187±6.34	0.01*
BMI (kg/m^2^)	24.88±4.32	24.97±4.53	27.01±4.16	0.24
Systolic BP (mmHg)	123.63±11.82	125.24±15.16	132.16±18.12	0.09
Diastolic BP (mmHg)	76.38±6.607	75.13±9.93	77.84±8.54	0.35
Biochemical parameters				
FBS (mg/dL)	175.1±62.17	197.07±72.26	201.56±71.21	<0.01*
HbA1C (%)	8.589±1.98	9.74±2.50	9.88± 2.97	<0.001*
Total cholesterol (mg/dL)	193.68±52.18	183.95±51.17	200.59± 50.50	0.03*
TGL (mg/dL)	175.86±83.56	157.07±84.55	201.65 ±138.68	0.01*
HDL-C (mg/dL)	42.85±16.88	40.6±12.48	41.45 ± 12.48	0.08
LDL-C (mg/dL)	124.833±45.40	121.46±44.90	131.84 ± 44.59	0.07
BUN (mmol/L)	11.72±4.26	13.8±8.79	20.74±13.08	0.01*
Creatine (սmol/L)	0.89±0.31	1.15±0.90	2.31±2.32	0.01*
VEGF-A (ng/mL)	1.61 ±1.57	2.75± 2.39	4.68±2.56	<0.001*
ANGPTL2 (ng/mL)	4.94±1.60	5.78±2.19	7.79± 2.03	<0.001*

According to our findings, serum VEGF-A exhibited statistical differences along the severity of disease (1.61 ±1.57 ng/mL in T2DM without DR, 2.75± 2.39 ng/mL in NPDR group, and 4.68± 2.56 ng/mL in PDR group, p<0.001*), and ANGPTL2 also exhibited a significant association between patients who had diabetic retinopathy and those who did not (T2DM without DR: 4.94±1.60 ng/mL, NPDR = 5.78±2.19ng/mL, PDR = 7.79±2.03ng/mL, p <0.001*).

To predict independent risk factors for NPDR and PDR

Collinearity was assessed by calculating the VIF. Only those variables that were <5 were included in the model. The status of PDR/NPDR was associated with ANGPTL2 after adjustment of risk factors; statistical significance was seen (p-value <0.001). Tables 2-3 show the multiple linear regression model for biomarkers and clinical variables. 

**Table 2:** Multiple linear regression model for VEGF-A and clinical variables *p<0.05 is statistically significant VIF: variance inflation factor; HDL: high-density lipoprotein cholesterol; TGL:  triglyceride level; NPDR: non-proliferative diabetic retinopathy; PDR: proliferative diabetic retinopathy

Variables	Β	p-value	VIF
HDL	-0.21	0.01*	2.08
TGL	0.07	0.98	1.32
Status of NPDR/ PDR	0.49	<0.001*	1.03

**Table 3:** Multiple linear regression model for ANGPTL2 and clinical variables *p<0.05 is statistically significant VIF: variance inflation factor; HDL: high-density lipoprotein cholesterol; TGL:  triglyceride level; NPDR: non-proliferative diabetic retinopathy; PDR: proliferative diabetic retinopathy

Variable	Β	p-value	VIF
HDL	-0.08	0.15	1.02
TGL	-0.001	0.98	1.03
Status of PDR/NPDR	0.51	<0.001*	1.00

The logistic regression analyses were performed to assess the presence of an independent association between markers and the risk of DR. To elucidate the independent effect of VEGF-A and ANGPTL2 on the presence of NPDR, binary logistic regression was done (Table 4). In the model, after adjustment with covariates age, sex, and years of diabetes mellitus, the results suggested that VEGF-A was an independent risk factor for NPDR.

**Table 4:** Binary logistic regression by adjusting covariates and detection of the independent effect of risk factors VEGF and ANGPTL2 on NPDR *p<0.05 is statistically significant SE: standard error; OR: odds ratio; T2DM: type 2 diabetes mellitus; NPDR: non-proliferative diabetic retinopathy

Variables	β	SE	OR (95% CI)	p-value
Age	0.06	0.01	1.01 (0.99-1.23)	0.06
Sex	0.52	1.10	0.65 (0.23-1.23)	0.75
Years of T2DM	1.21	0.09	1.54(1.31-1.94)	0.01*
VEGF-A (ng/mL)	0.97	0.12	0.72 (0.59-0.97)	0.001*
ANGPTL2 (ng/mL)	1.02	0.125	0.78 (0.61-0.97)	0.001*

To elucidate the independent effect of VEGF-A and ANGPTL2 on the presence of PDR, binary logistic regression was done. In the model, after adjustment with covariates age, sex, years of diabetes mellitus, and HbA1c, the results suggested that VEGF-A was an independent risk factor for PDR (Table 5).

**Table 5:** Binary logistic regression by adjusting covariates and detection of the independent effect of risk factors VEGF and ANGPTL2 on PDR *p<0.05 is statistically significant SE: standard error; OR: odds ratio; T2DM: type 2 diabetes mellitus; PDR: proliferative diabetic retinopathy

Variables	Β	S.E	OR (95% CI)	p-value
Age	0.04	0.02	1.04 (0.99-1.41)	0.06
Sex	0.41	1.32	0.65 (0.3-1.44)	0.75
Years of T2DM	1.31	0.13	0.73(1.56-1.945)	0.01*
VEGF-A (ng/mL)	1.11	0.48	1.55 (1.11-1.73)	<0.001*
ANGPTL2 (ng/mL)	1.22	0.32	1.54 (1.40-2.44)	0.001*

Correlation between markers in all three groups

**Figure 1:**
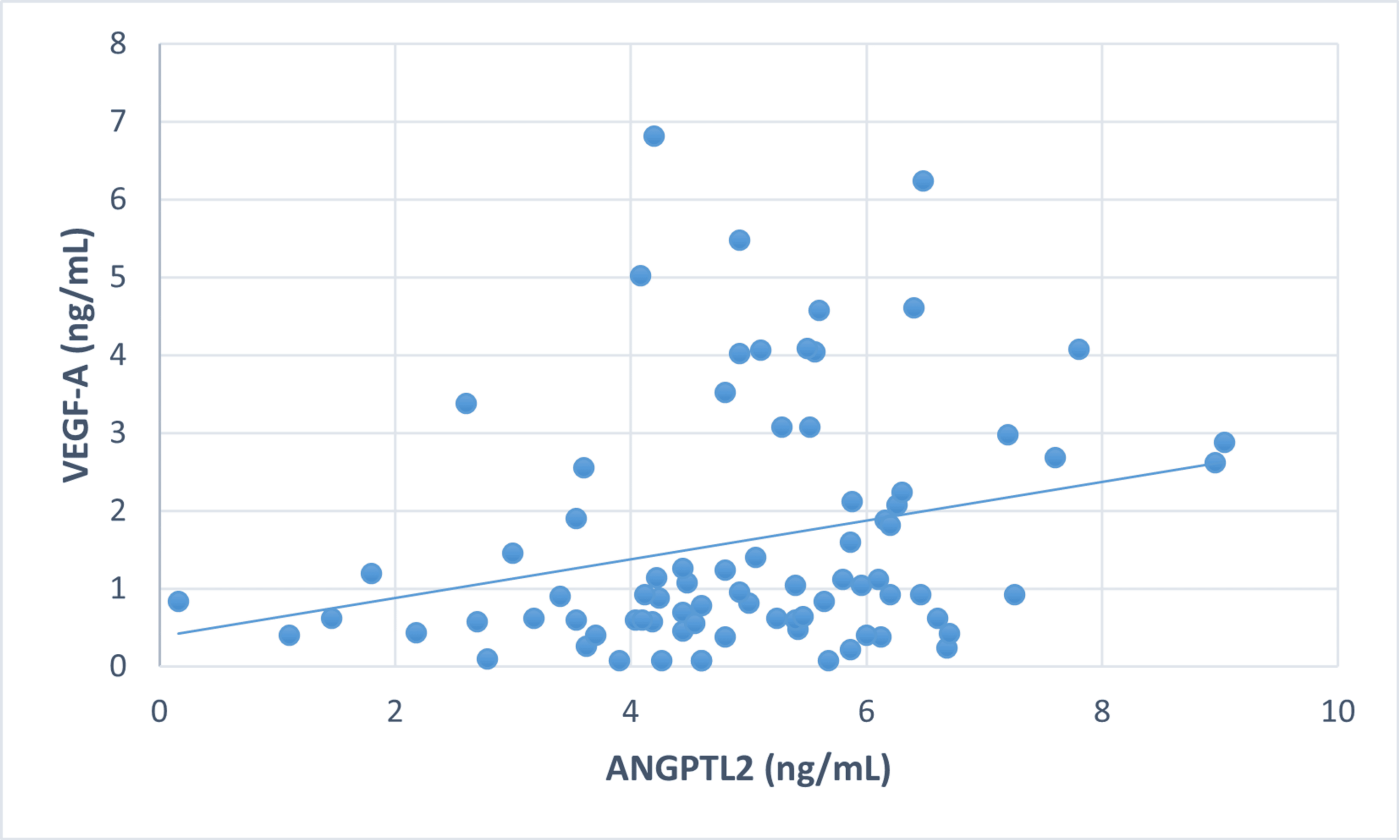
Correlation between VEGF-A and ANGPTL2 in T2DM without diabetic retinopathy T2DM: type 2 diabetes mellitus

**Figure 2:**
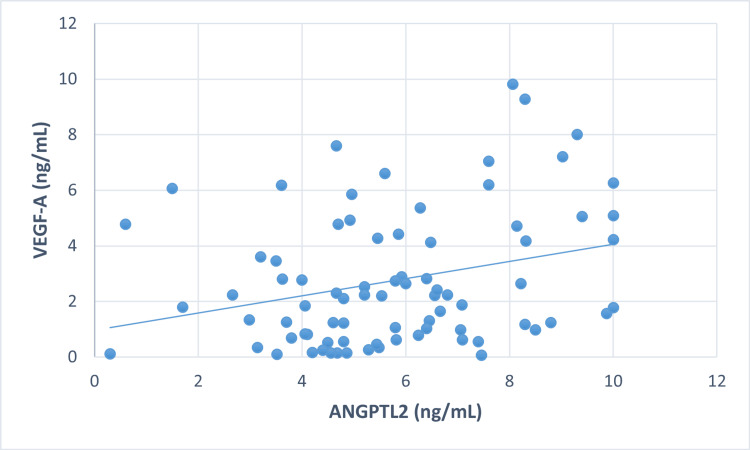
Correlation between VEGF-A and ANGPTL2 in non-proliferative diabetic retinopathy

**Figure 3:**
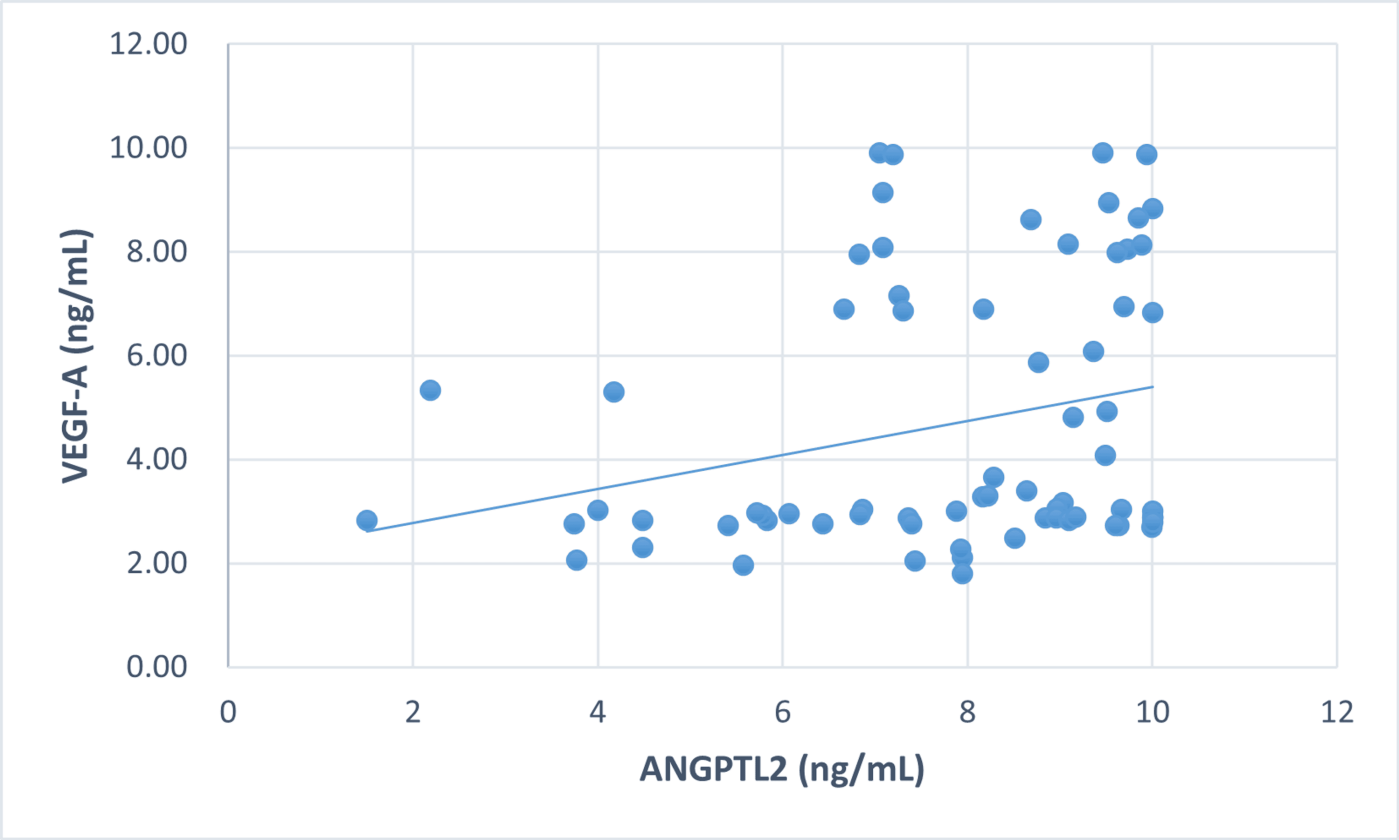
Correlation between VEGF-A, and ANGPTL2 in proliferative diabetic retinopathy

To explore the relationship between VEGF-A and ANGPTL2 in all groups, correlation analysis was applied (Figures 1-3).

A significant positive correlation was observed between VEGF-A and ANGPTL2 in control (r = 0.253, p<0.05), NPDR (r = 0.284, p<0.05), and PDR (r = 0.259, p<0.05). The elevated VEGF-A levels were significantly correlated with FBS, the primary cause of diabetes, in all three groups: control (r = 0.226, p<0.05*), NPDR (r = 0.247, p<0.05*), and PDR (r = 0.231, p<0.05*). Additionally, in the NPDR group, there was a significant correlation between serum TC (r = 0.407, p<0.001*) and LDL-C (r = 0.290, p<0.05*). However, no correlation was found between ANGPTL2 and other parameters, such as FBS and lipid profile.

Diagnostic performance of biomarkers for prediction of NPDR and PDR in T2DM

**Figure 4:**
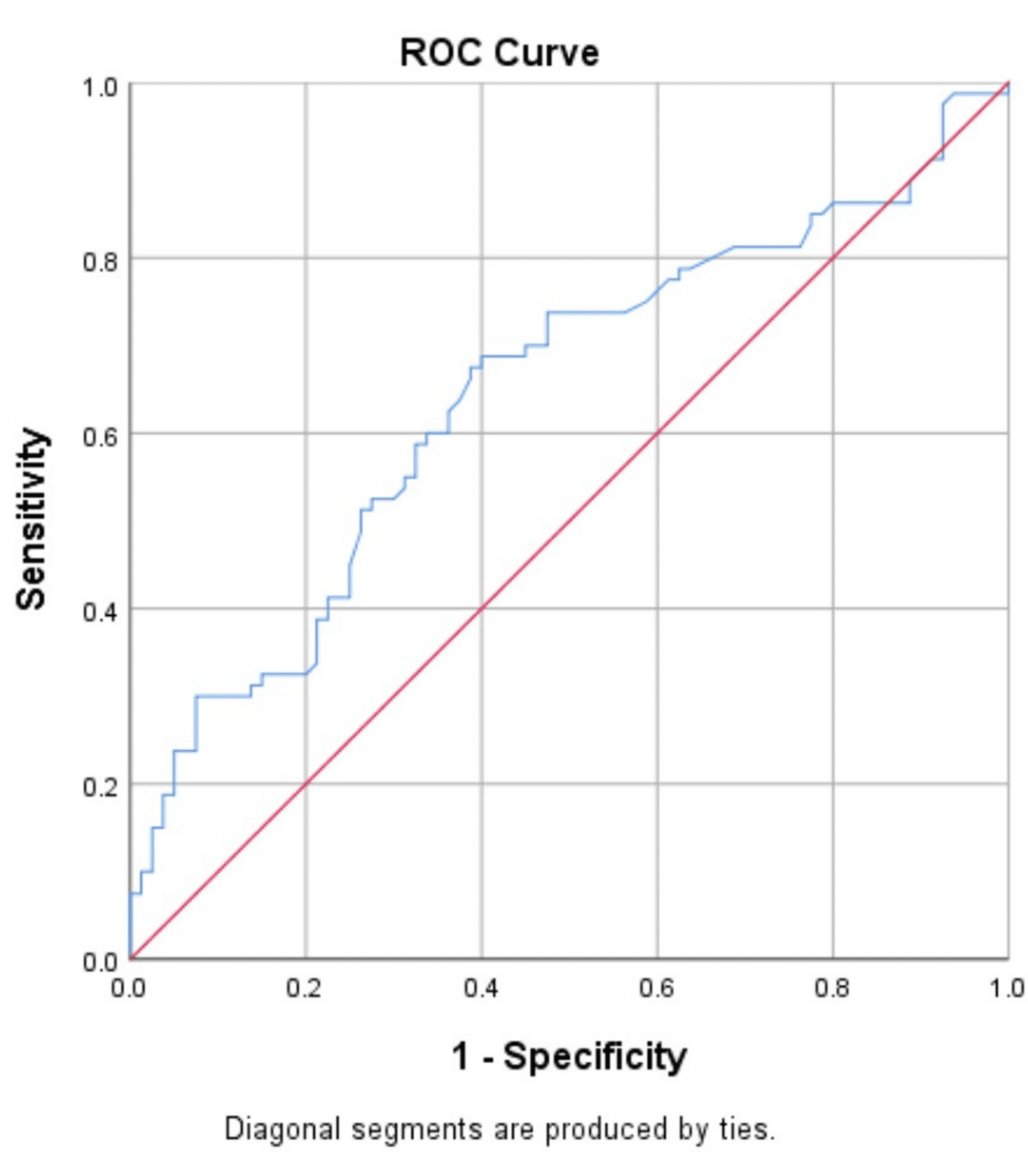
Receiver operating characteristic (ROC) analyses of the predictive efficacy of VEGF-A in the diagnosis of non-proliferative diabetic retinopathy

**Figure 5:**
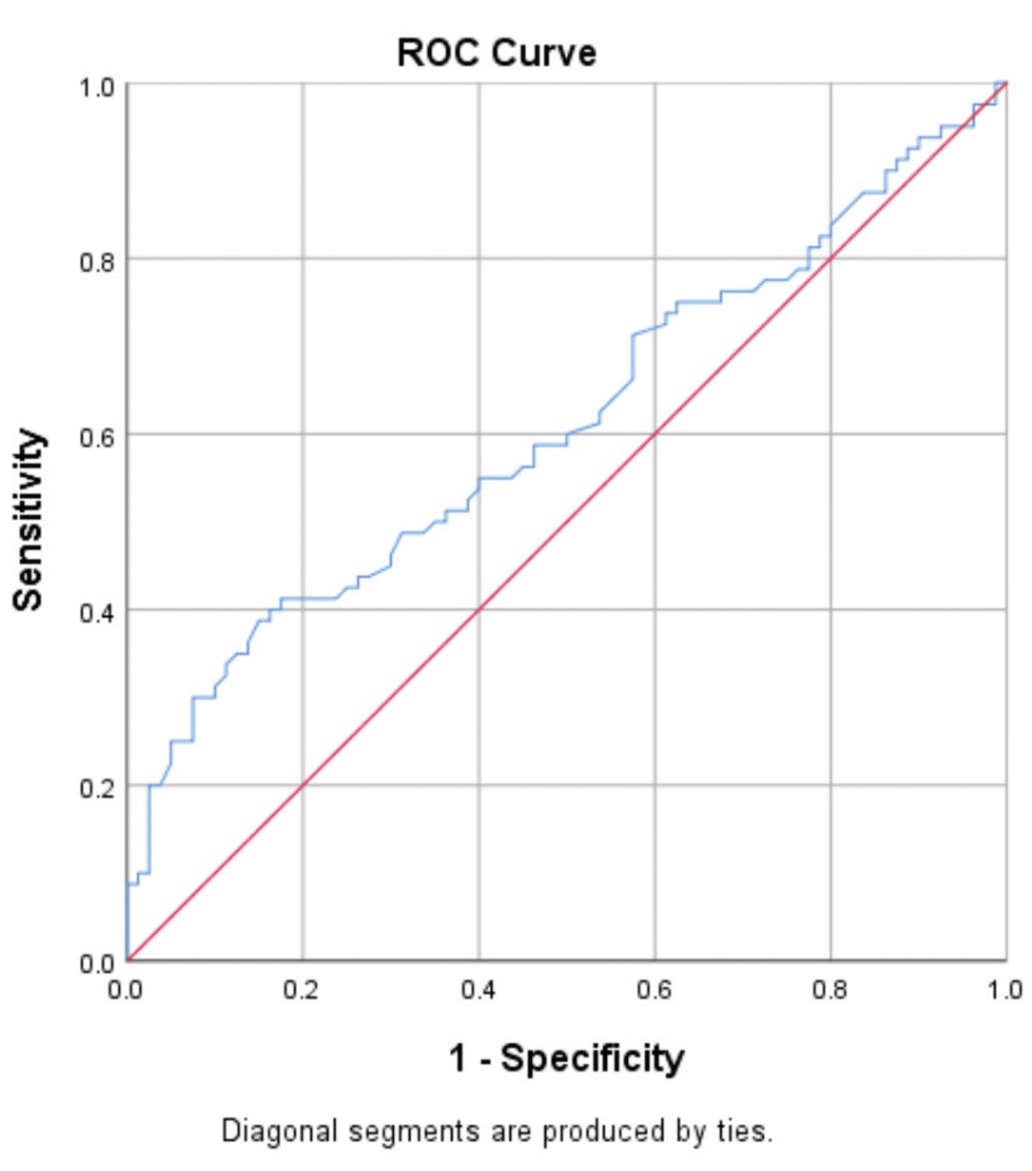
Receiver operating characteristic (ROC) analyses of the predictive efficacy of ANGPTL2 in the diagnosis of non-proliferative diabetic retinopathy

**Figure 6:**
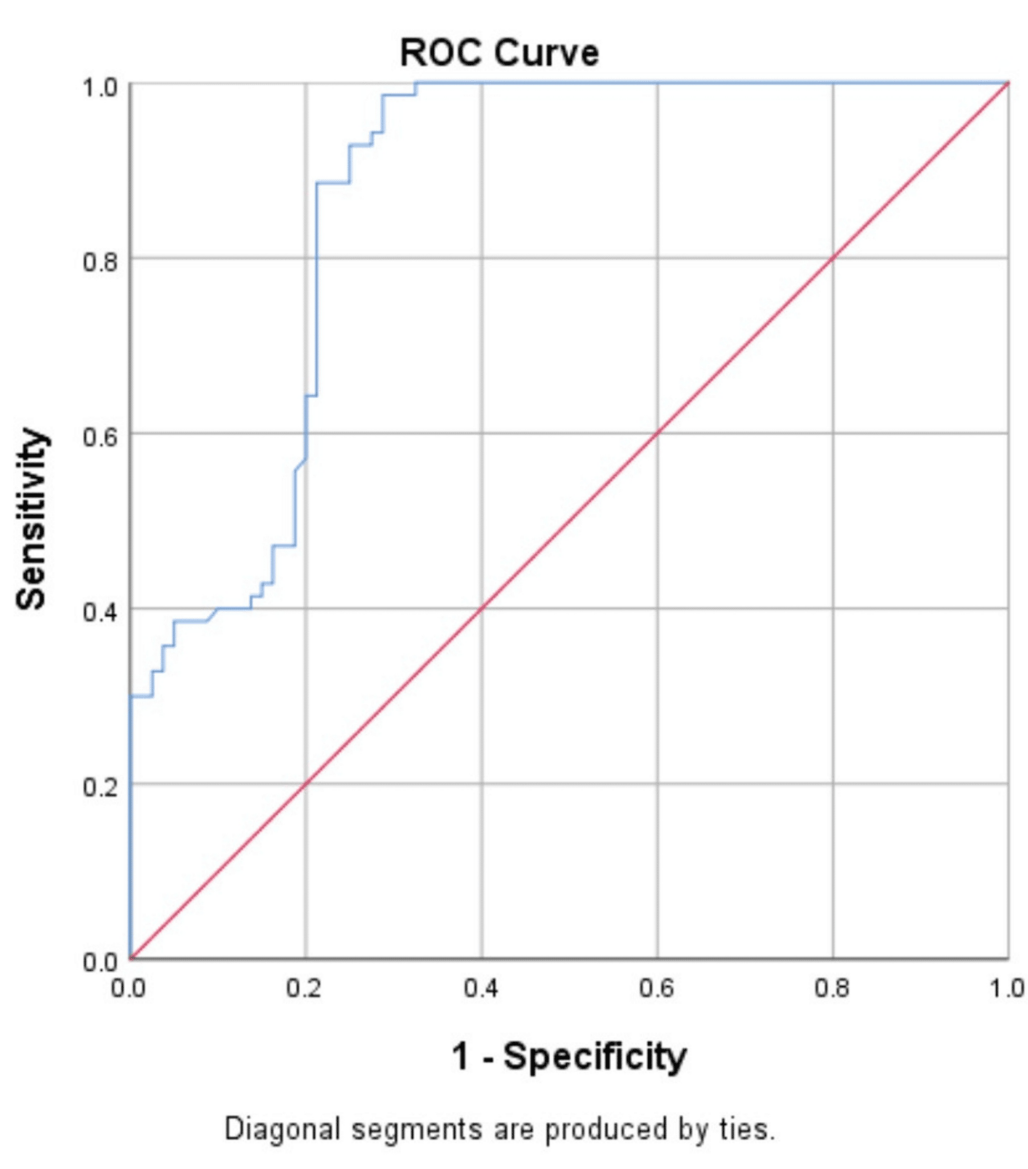
Receiver operating characteristic (ROC) analyses of the predictive efficacy of VEGF-A in the diagnosis of PDR

**Figure 7:**
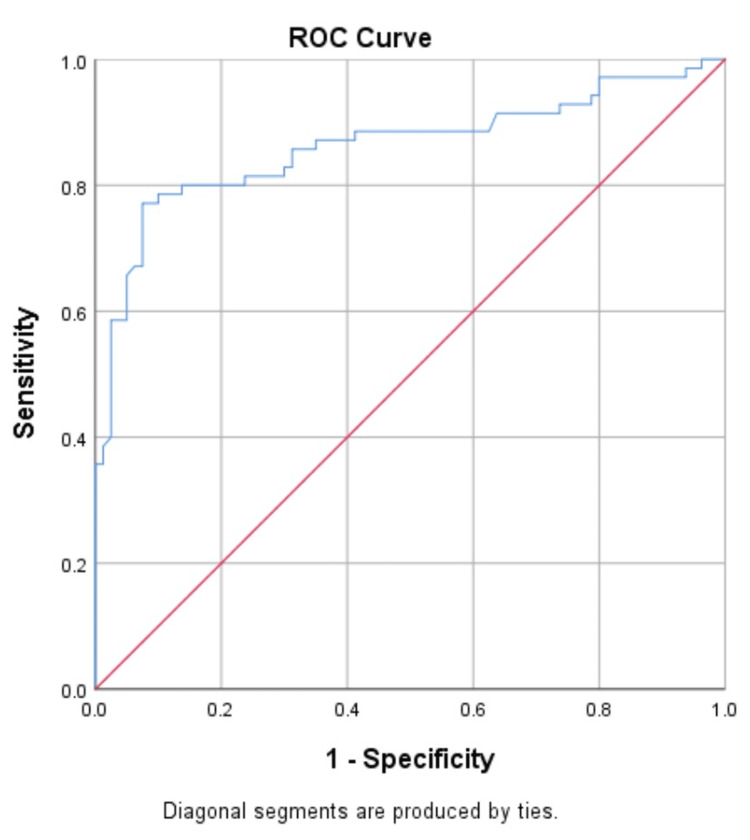
Receiver operating characteristic (ROC) analyses of the predictive efficacy of ANGPTL2 in the diagnosis of PDR

The ROC curve was constructed to determine the best threshold value for distinguishing patients with NPDR and PDR (Figures 4-7). 

The ROC indicated that VEGF-A and ANGPTL2 act as risk biomarkers for the prediction of proliferative diabetic retinopathy with an area under the curve of 0.86 (p-value<0.001) when compared with control (T2DM without DR) (Tables 6, 7) and found the optimal cut-off value with the best diagnostic performance.

**Table 6:** Sensitivity and specificity of VEGF-A and ANGPTL2 for the prediction of NPDR NPDR: non-proliferative diabetic retinopathy

	VEGF-A	ANGPTL2
Sensitivity	63.75%	62.5%
Specificity	57.51%	46.25%
Youden’s index	0.21	0.08
Positive predictive value (PPV)	60%	53.76%
Negative predictive value (NPV)	61.33%	55.2%
Area under the curve (AUC)	0.62	0.61
Cut-off	>1.07	>4.89

**Table 7:** Sensitivity and specificity of VEGF-A and ANGPTL2 for the prediction of proliferative diabetic retinopathy

	VEGF-A	ANGPTL2
Sensitivity	98.52%	90.9%
Specificity	96.34%	88.09%
Youden’s index	0.94	0.78
False negative rate	1.48%	9.1%
False positive rate	3.6%	11.91%
Area under the curve (AUC)	0.86	0.86
Cut off	>2.29	>5.58

## Discussion

The key findings of this study are summarized as follows: (1) serum VEGF-A and ANGPTL2 levels were higher in the DR group compared with the control group; (2) a significant correlation was observed between these markers.

A former study was done on serum ANGPTL2 in diabetic retinopathy patients with and without macular edema. In this study, an increasing trend was found between the control (T2DM), non-clinically significant macular edema (nCSME) group, and clinically significant macular edema (CSME) group. The mean of ANGPTL2 was 4.46 ng/mL in the CSME, 3.80 ng/mL in the nCSME group, and 3.33 ng/mL in the control [13], which is similar to our results, where we found that serum ANGPTL2 levels are significantly higher in cases of NPDR and PDR as compared to T2DM without DR. After covariates like age, sex, years of diabetes mellitus, and HbA1C were adjusted for in the model, a binary logistic regression was conducted. The outcomes confirmed that ANGPTL2 is a distinct risk factor for PDR. This marker was also elevated in other microvascular diseases, like cardiovascular disease. According to a study by El-Lebedy [14], it was significantly higher in T2DM with cardiovascular disease (CVD) than in T2DM without CVD and healthy controls. Additionally, FBS, hbA1C, insulin, Homeostatic Model Assessment for Insulin Resistance (HOMA-IR), total cholesterol, TGL, and LDL-C were also found to be statistically significant. Similarly, patients with diabetic foot ulcer (DFU) exhibited elevated ANGPTL2 levels when compared to subjects with T2DM only (T2DM vs. DFU: 4.221 ± 1.301 vs. 6.561 ± 2.335 μg/L, p < 0.0001) [15]. These studies suggest that this marker, which is more prevalent in T2DM than in healthy people, contributes to microvascular illness. As prior research is sufficient on healthy participants and T2DM, we did not include healthy as a control here.

In addition, our investigation demonstrated a strong correlation between the degree of diabetic retinopathy and elevated VEGF-A. The mean levels were 1.614±1.57 ng/mL, 2.75±2.39 ng/mL, and 4.68±2.56 ng/mL in the control, NPDR, and PDR groups, respectively. A binary logistic regression was conducted to clarify the independent impact of VEGF on the presence of PDR. The results of the model's adjustment with covariates such as age, sex, years of diabetes mellitus, and HbA1c indicated that it functions as a separate risk factor for PDR. It was observed that the presence of inadequate glycemic control raised the level of this marker. Our results were consistent with two studies: According to Ahuja et al., there was a significant upregulation of serum VEGF-A, with an area under the curve (AUC) of 0.761±0.056 between NPDR and PDR, from 138.96±63.37 pg/ml (controls) to 457.18±165.69 pg/ml (PDR) (F = 48.47; p<0.001) [16]. Also, DR was positively correlated with urea, creatinine, and the length of diabetes. Another study by Unung [17] found a significant difference in systolic (P = 0.0001) and diastolic blood pressure (BP) (P = 0.001), fasting plasma glucose, HbA1c, TC, TGL, HDL-C, VEGF, and pigment epithelium-derived factor (PEDF). Similarly, Keles [18] investigated the association of this marker on a vitreous sample and reported that there was a significant positive correlation in vitreous levels of SDF-1α in a patient with active PDR (r = 0.545, p = 0.002), but no significant correlation was observed between the vitreous levels of ANGPTL2 and VEGF (r = 0.271; p = 0.141).

In contrast to that study, we found a significant positive correlation between VEGF-A and ANGPTL2 in the serum of diabetic patients with and without diabetic retinopathy. Both markers showed good diagnostic values, with an AUC of 0.86. The advantage of our work is that, while previous research on vitreous samples has demonstrated a positive and significant correlation, those markers require invasive procedures by skilled ophthalmologists, whereas serum analysis is inexpensive and non-invasive.

Limitations

As the study is cross-sectional, longitudinal cohort studies are necessary to validate the results. The ethnicity of the participants is not taken into consideration because the study is hospital-based and the inclusion of participants is from tertiary care.

## Conclusions

Our data demonstrate that higher levels of circulating serum ANGPTL2 and VEGF-A may be associated with DR in patients with T2DM. Additionally, a strong positive correlation was observed between them. The elevated serum markers in DR may indicate a potential new therapeutic agent or serve as a predictive and prognostic indicator during the proliferative stage of development. Further prospective studies are needed to reveal its role in advanced retinal disease.
